# Menstrual practice needs scale for the workplace: validation and associations with well-being among adult women in Kathmandu, Nepal and Nairobi, Kenya

**DOI:** 10.3389/fgwh.2026.1693187

**Published:** 2026-04-22

**Authors:** Allison P. Salinger, Regine Haardörfer, Julie Hennegan, Madeleine Patrick, Amelia Conrad, Anupama Ramaswamy, Aparna Stephen, Sheela S. Sinharoy, Bethany A. Caruso

**Affiliations:** 1 Hubert Department of Global Health, Rollins School of Public Health, Emory University, Atlanta, GA, United States; 2 Behavioral, Social, and Health Education Sciences Department, Rollins School of Public Health, Emory University, Atlanta, GA, United States; 3 Maternal, Child and Adolescent Health Program, Burnet Institute, Melbourne, VIC, Australia; 4 School of Public Health, University of Queensland, Brisbane, BNE, Australia; 5 Athena Infonomics, Chennai, India

**Keywords:** economic empowerment, instrument development, menstrual health, menstrual practice needs scale, mental health, validation, workplace

## Abstract

**Introduction:**

Unmet menstruation-related needs are common among women in low- and middle-income settings, negatively affect physical and mental health, and limit participation in education and employment. The primary focus of research on menstrual health (MH) has been adolescent girls. Menstrual experiences of adult women, especially in the workplace, are understudied.

**Materials and methods:**

This study validated an adapted tool for measuring menstrual experiences in the workplace (MPNS-W) and assessed relationships between MPNS-W scores and well-being outcomes among 892 working women in urban settings in Kenya and Nepal. We used exploratory and confirmatory factor analyses to test validity and dimensionality of the MPNS-W. Construct validity was assessed using binary logistic regressions of MPNS-W scores on *a priori* selected well-being measures.

**Results:**

Factor analyses yielded a solution with good model fit. The model has four factors: workplace-related menstrual material satisfaction and access, disposal and changing environment, transport and storage, and menstrual material reliability. Tests of measurement invariance confirmed the MPNS-W performed equivalently across settings, and tests of internal consistency demonstrated its reliability. Controlling for wealth score, women with higher MPNS-W scores had significantly higher odds of reporting positive psychological well-being, not missing work because of the last menstrual period, and being completely confident managing menstruation at work.

**Discussion:**

MPNS-W scale scores can be used to assess determinants and outcomes of MH in the workplace while sub-scale scores can be examined relative to one another to identify priorities. Menstrual needs of women in the workplace are multidimensional and strongly associated with well-being, making tools like the MPNS-W critical for informing and evaluating programs designed to address menstrual experiences in the workplace.

## 1 Introduction

Negative experiences of menstruation and unmet menstruation-related needs are common among women in low- and middle-income country (LMIC) contexts (1). Lack of access to private and safe water and sanitation facilities for changing, washing, and disposing materials as needed (2, 3); constrained availability and accessibility of preferred, affordable, and reliable materials (4, 5); limited practical knowledge about how to effectively catch or absorb menstrual blood and manage pain; and unsupportive social environments and stigma all pose major barriers to women's ability to meet their needs for menstrual health (MH) (1, 6), which is defined as “a state of complete physical, mental, and social well-being and not merely the absence of disease or infirmity, in relation to the menstrual cycle” (7). The impacts of these negative menstruation experiences and unmet needs include negative health outcomes such as reproductive tract infections (8) and higher rates of self-reported urogenital symptoms (9); impacts on mental well-being in the form of increased stress, anxiety, and depression (10, 11); impacts on social well-being including social isolation (12); and limited ability to participate in education and employment (11).

While research, programming, and discourse for menstrual health in LMICs continue to gain attention globally (13–17), the focus of much of this work has been on adolescents, particularly on school-going girls (3, 18, 19). A systematic review and meta-synthesis of studies reporting qualitative analyses of menstrual experiences among women and girls in LMICs identified more than three times as many studies of adolescent school-aged girls (*n* = 55) than of adult women exclusively (*n* = 16) (1). Similarly, instrument development has focused on constructing and validating tools among populations of adolescent girls (20–22). Experiences of menstruation among adult women are understudied by comparison (6), especially among those who experience menstruation in the workplace (23, 24). The few studies that have focused on this population find that between 11% and 36% of women surveyed in Burkina Faso, Niger, Nigeria, and Ghana reported missing work due to menstruation (23, 25). Unmet menstruation-related needs can negatively impact women's earnings and advancement opportunities in the short-term (5). If unresolved, these challenges can act as barriers to women's long-term economic empowerment and gender equality in the workforce (26, 27).

The Advancement of Metrics for Menstrual Health and Hygiene in the Workplace activity under the Water, Sanitation and Hygiene Partnerships and Learning for Sustainability (WASHPaLS) project was designed to help fill this gap (26). The activity, led by Emory University and Athena Infonomics, aimed to test new and adapt existing MH measurement tools and recommend indicators for assessment of experiences, determinants, and impacts related to menstruation in the workplace. The team adapted the Menstrual Practice Needs Scale (MPNS) to assess menstruation practices and needs specific to the workplace (26). For the purpose of this study, “workplace” includes any location outside the home where participants conducted work for cash or in-kind payment; workplaces may be shifting (e.g., street vendors), or fixed (e.g., office workers) and formal or informal.

The MPNS is based on findings from a systematic review and meta-synthesis of qualitative studies from LMICs (1) and a survey of experts (21). The creators of the tool argue for the need to move beyond measures of quantity or type of menstrual materials to measures that allow for practices and the environment to be appraised differently by women from different sociocultural contexts; hence, the MPNS aims to “*capture the extent to which respondents', current menstrual management practices and environments are perceived to meet their needs”* (21)*.* The MPNS was initially validated among a population of adolescent schoolgirls in Uganda (21) and subsequently adapted and revalidated among a population of adult working women in Uganda (23). The revalidation found that the concepts measured among adult working women were different from those among adolescent schoolgirls; changes were required to validate the scale among the population of adult working women (23). The sample population was also limited to women working in markets, schools, or healthcare facilities only.

For these reasons, there is the need for a validated tool for women from other cultural contexts and work environments. Additionally, menstrual practices have “location dependency”: a study in Bangladesh found that schoolgirls' menstrual practices and confidence to manage menstruation were different in the home environment versus at school (28). Previous iterations of the MPNS include items that refer to the home and school or to the home and work environments (21, 23). This study aims to validate an adapted version of the MPNS that will exclusively measure menstrual experiences in the workplace (MPNS-W). We investigated the performance of this measure among adult women working outside the home in a variety of work environments in two new country contexts: urban areas of Nairobi, Kenya and Kathmandu, Nepal. As part of the validation, we also explore associations between MPNS-W scores and key well-being measures including psychological well-being, menstruation-related self-efficacy, and work absenteeism, controlling for wealth.

## 2 Materials and methods

This was a cross-sectional study using survey data from the Advancement of Metrics for Menstrual Health and Hygiene in the Workplace activity under the WASHPaLS project, which was implemented across two cities (Kathmandu, Nepal and Nairobi, Kenya) in September and October, 2021.

### 2.1 Participants

Sample size was calculated based on recommendations for scale validation, which suggest 5 to 15 respondents per item (29, 30). After cognitive interviews and adaptations to focus exclusively on the workplace environment (i.e., excluding items related to the home environment), we retained 28 of the original 36 MPNS items. We aimed to survey approximately 600 women in each country to allow for non-response and missing data.

In Kenya, Adaptive Management and Research Consultants (AMREC, the local data collection firm) and in-country experts selected 23 urban neighborhoods from among four sub-counties (10 in Embakasi, 3 in Kasarani, 6 in Njiru, and 4 in Lang’ata). In Nepal, the Nepal Institute for Social and Environmental Research (NISER) and in-country experts selected 6 urban neighborhoods from among two districts (3 in Kathmandu and 3 in Lalitpur). Sub-counties, districts, and neighborhoods were selected, based on discussions with country experts, to maximize variation in job type, work sector, and socioeconomic status. Households were selected using systematic random sampling whereby enumerators knocked on every third door. If there was no answer, the enumerator proceeded to the next immediate house. If a woman answered, but was ineligible or refused to participate, the enumerator proceeded with the random sampling approach (i.e., moved three doors down).

Women were eligible for participation in the survey if they were at least 18 years of age, had experienced a menstrual period while working outside their homes for cash or in-kind payments in the previous three (Kenya) or six (Nepal) months, passed a COVID-19 screening questionnaire (including a temperature check and self-reported exposure and symptoms), and were able to verbally communicate in English, Swahili, or Nepali. The timeframe for experiencing a menstrual period while at work was expanded from three to six months in Nepal due to persistent COVID-19 lockdowns.

### 2.2 Data collection

The full survey tool was refined based on (1) review by subject matter experts in the field of menstruation and measurement, (2) feedback from stakeholders including local institutional review boards and data collection teams, and (3) findings from cognitive interviews with 15 women field staff in Kenya and 12 women field staff in Nepal. Additional information about the full survey and cognitive interview procedures and findings are detailed elsewhere (26). These refinement steps were taken to test and ensure appropriateness (content validity) and interpretability (face validity) of the survey items (31). The current analysis focuses on assessing scale dimensionality, measurement invariance, internal consistency, and construct validity. The survey was translated from English into Swahili and Nepali and independently back-translated, and cognitive interviews were used to further refine translation. The survey tool was piloted in Kenya and Nepal where each enumerator conducted two to three pilot surveys on average. Surveys were programmed using Open Data Kit (ODK).

Trained enumerators (managed by AMREC in Kenya and NISER in Nepal) administered surveys on Android tablets between September and October 2021. Surveys took between approximately 45 and 90 minutes, depending on skip patterns triggered by women's responses, and were conducted in women's homes, except in cases in Nepal where some self-employed women (who were recruited at their homes) requested to be interviewed at their workplaces. Surveys were conducted in Swahili, Nepali, or English depending on the respondents' preference.

### 2.3 Measures

#### 2.3.1 Menstrual Practice Needs Scale

To understand experiences and practices related to menstruation in the workplace, we used the MPNS-W, an adapted version of the MPNS (23). The MPNS-W section of the survey advised participants to “*Please think about the last menstrual period you had while working at your main job outside the home*” to inform their response to each item. For each item, response options were “never”, “less than half the time”, “more than half the time”, and “always.” We followed the procedure described in Hennegan et al. (11) for scoring the MPNS: positively oriented items (e.g., “*Were the materials you used to absorb or catch menstrual blood comfortable*?”) were scored from 0 (never) to 3 (always). Negatively oriented items (e.g., “*Were you worried about how you would get more of your menstrual material if you ran out*?”) were scored from 3 (never) to 0 (always). MPNS-W scale scores and sub-scale scores were calculated as a mean for all relevant items such that higher scores reflect more positive menstruation experiences (possible range: 0–3). In order to better explore relationships with well-being measures of interest, these MPNS-W scale scores were subsequently categorized as follows: needs met *always* (MPNS-W scale score = 3), needs met *more than half the time* (MPNS-W scale score <3 and ≥2), needs met *less than half the time* (MPNS-W scale score <2 and ≥1), and needs met *never* (MPNS-W scale score <1).

As our focus was on the workplace only, we omitted some household-related items from the version of the MPNS (36 items) that was validated among adult women in Uganda. Specifically, four items are duplicated in the MPNS to ask about the same experience separately “*when at home*” (items 17 and 19–21 in the MPNS) versus “*when at work*” (items 24 and 26–28 in the MPNS; items 17 and 19–21 in the current study). We omitted the four “duplicate” items that explicitly refer to the home environment. Two items “*Were you able to dispose of your used materials in the way that you wanted to?”* (item 13) and “*Were you concerned that others would see your used menstrual materials in the place you disposed of them?*” (item 15) were found to be unclear and/or irrelevant to respondents in the study contexts during cognitive interviews and were therefore omitted. The MPNS-W and all revisions made as part of the adaptation and survey refinement process are documented in Supplementary Table S1.

#### 2.3.2 Wealth index

To assess wealth, we generated an index by summing binary responses (yes/no) regarding household ownership of 22 assets (e.g., radio, television, refrigerator). The lists of assets were taken from country-specific questions in the Demographic and Health Surveys and sums standardized for comparability and subsequently converted into wealth quintiles.

#### 2.3.3 Psychological well-being

To assess psychological well-being, we used the World Health Organization (WHO)-5 Well-Being Scale, a widely validated measure that includes five items concerning the frequency of specific feelings over the previous two weeks. For example, “*Over the last two weeks, I have felt calm and relaxed.*” Response options include “at no time”, “some of the time”, “less than half of the time”, “more than half of the time”, “most of the time”, and “all the time” (scored 0 to 5, respectively). Responses are summed such that sum scores range from 0 to 25; individuals with scores below 13 are considered to have poor well-being (32, 33).

#### 2.3.4 Menstruation-related self-efficacy

To assess confidence to manage menstruation, or menstruation-related self-efficacy, the following survey item was used: “*Managing menstruation at work can involve changing, washing, disposing of materials, and other behaviors. How confident do you feel in your ability to manage your menstruation when working outside the home?”* Response options included “not at all confident”, “slightly confident”, “very confident”, and “completely confident”. We dichotomized this measure (for consistency across other binary well-being measures and to improve interpretability) such that respondents were categorized as either “completely confident” or “not completely confident” (including all who responded, “not at all confident”, “slightly confident”, or “very confident”).

#### 2.3.5 Work absenteeism

To assess work absenteeism, participants were asked whether they “*missed an entire day of work because of last menstruation*” or “*missed a partial day of work/some hours of work because of last menstruation*.” These were combined such that women who responded yes to at least one of the two items were categorized as having “missed work” and women who responded no to both were categorized as “did not miss work.”.

### 2.4 Analysis

Factor analysis is used to assess the hypothesized relationships between observed variables and their underlying latent constructs (34). We conduct factor analyses to identify the dimensionality of the MPNS-W (i.e., to determine how MPNS-W items co-vary and define the constructs underlying these relationships). We modeled our methodology for assessing dimensionality, internal consistency, and construct validity (34, 35) of the MPNS-W on Hennegan et al. (11), in which the creators of the MPNS revalidated the scale for adult working women in Uganda. In keeping with Hennegan et al. (11), the analytic sample included only those women who reported changing menstrual materials while working outside the home during their last menstrual period. Hennegan et al. (11) assessed dimensionality, internal consistency, and construct validity of the MPNS among women who dispose of menstrual materials and women who reuse menstrual materials separately, as there are several items in the scale that are only applicable to women who use reusable menstrual materials. However, less than 8% of women (75 of 940 who changed menstrual materials at work during their last period) in our sample reported ever using reusable menstrual materials while working. Our examination of item distributions (as described below) also identified substantial missingness across the reuse-related items (items 29–38 and 44) (Supplementary Table S2). Therefore, our analytic sample includes only those women who reported disposing of menstrual materials at work (N_Dispose_ = 892). The analytic sample of women is described in Table 1 in terms of their demographic information (e.g., age, marital status, education) employment information (e.g., job type and job stability of main job) and menstruation practices (e.g., type of material used, change and disposal locations) overall and by country.

**Table 1:** Demographic and employment information and menstruation practices, by country.

Participant characteristic	All (*N* = 892)		Kenya (*N* = 565)		Nepal (*N* = 327)	
Age (Mean, Range)	30.0	18–53	29.3	18–53	31.2	18–49
	*N*	%	*N*	%	*N*	%
Marital status						
Single, never married	362	40.6%	221	39.1%	141	43.3%
Married	420	47.1%	252	44.6%	168	51.5%
Unmarried, living with partner	48	5.4%	48	8.5%	0	0%
Divorced/separated	41	4.6%	33	5.8%	8	2.5%
Widowed	20	2.2%	11	2.0%	9	2.8%
Education						
Less than primary education	14	1.6%	3	0.5%	11	3.4%
Primary education	28	3.2%	20	3.6%	8	2.5%
Secondary education	250	28.2%	139	24.7%	111	34.1%
Above secondary education	595	67.0%	400	71.2%	195	59.8%
Other	1	0.1%	0	0%	1	0.3%
Wealth Quintile						
Quintile 1 (lowest)	166	18.7%	108	19.2%	58	18.0%
Quintile 2	192	21.7%	141	25.0%	51	15.8%
Quintile 3	168	18.9%	91	16.1%	77	23.8%
Quintile 4	187	21.1%	113	20.0%	74	22.9%
Quintile 5 (highest)	174	19.6%	111	19.7%	63	19.5%
Job Type (main job)						
Professional/office work (e.g., financial services, IT, research)	192	21.6%	111	19.7%	81	24.8%
Working in a shop or store (retail)	146	16.4%	88	15.6%	58	17.7%
Food or lodging (e.g., restaurant, hotel)	95	10.7%	54	9.6%	41	12.5%
Selling goods in a marketplace, street, or other informal setting	87	9.8%	76	13.5%	11	3.4%
Teaching/education/tutoring	87	9.8%	49	8.7%	38	11.6%
Factory/manufacturing/textiles	41	4.6%	26	4.6%	15	4.6%
Health care worker	80	9.0%	54	9.6%	26	8.0%
Civil servant/government employee	57	6.4%	34	6.0%	23	7.0%
Day labor/casual or informal labor (non-farming)	44	4.9%	37	6.6%	7	2.1%
Domestic work (e.g., cleaning homes)	37	4.2%	22	3.9%	15	4.6%
Other	15	1.7%	9	1.6%	6	1.8%
Farming/agriculture/forestry/fishing	10	1.1%	4	0.7%	6	1.8%
Job Stability (main job)						
Throughout the year	824	92.4%	506	89.6%	318	97.3%
Seasonally/part of the year	59	6.6%	53	9.4%	6	1.8%
Once in a while	9	1.0%	6	1.1%	3	0.9%
All materials used to catch/absorb blood while working outside the home during last menstrual period						
Single-use/disposable sanitary pads	812	91.0%	510	90.3%	302	92.4%
Tampons	72	8.1%	61	10.8%	11	3.4%
Cloth	56	6.3%	5	0.9%	51	15.6%
Reusable sanitary pads	53	5.9%	41	7.3%	12	3.7%
Toilet paper	32	3.6%	28	5.0%	4	1.2%
Menstrual cup	12	1.4%	5	0.9%	7	2.1%
Cotton wool	11	1.2%	10	1.8%	1	0.3%
Underwear alone	10	1.1%	10	1.8%	0	0
Absorbent underwear/panties	7	0.8%	2	0.4%	5	1.5%
Other	3	0.3%	3	0.5%	0	0
Place used to change menstrual materials most often while working outside the home						
Facility/toilet at my workplace	736	82.5%	427	75.6%	309	94.5%
Public/shared toilet outside my workplace	102	11.4%	98	17.4%	4	1.2%
Facility/toilet at another place of business	26	2.9%	19	3.4%	7	2.1%
Toilet at someone else's home	13	1.5%	10	1.8%	3	0.9%
Toilet at my home	7	0.8%	5	0.9%	2	0.6%
Use the outdoors/in the bush or a field (do not use a facility)	5	0.6%	4	0.7%	1	0.3%
Private room at my workplace (without toilet/latrine)	2	0.2%	2	0.4%	0	0%
Other	1	0.1%	0	0%	1	0.3%
Place used to dispose used menstrual materials most often while working outside the home						
Put in a rubbish bin inside toilet stall at workplace	453	51.0%	254	45.0%	199	61.2%
Put in latrine or toilet at workplace	184	20.7%	155	27.5%	29	8.9%
Put in a rubbish bin outside toilet stall at workplace	113	12.7%	55	9.8%	58	17.9%
Transported home to dispose or reuse	66	7.4%	44	7.8%	22	7.4%
Put in a community rubbish bin outside of workplace	56	6.3%	46	8.2%	10	3.1%
Other	9	1.0%	4	0.7%	5	1.5%
Threw or buried somewhere outside	8	0.9%	6	1.1%	2	0.6%

% is out of those who chose to answer: (Marital Status: 1 in Nepal chose not to answer; Education: 3 in Kenya and 1 in Nepal chose not to answer; Wealth Quintile: 1 in Kenya and 4 in Nepal missing; Job Type: 1 in Kenya chose not to answer; Place to dispose: 1 in Kenya and 2 in Nepal chose not to answer.) Menstrual materials: response options not selected by any respondents included natural material, mattress or foam, and no material.

#### 2.4.1 Exploratory factor analysis

The current study introduces not only new country contexts but also a number of adaptations to the scale to ensure applicability for menstruation experiences in the workplace (excluding the home environment). For these reasons, it is pertinent to start with exploratory factor analysis (EFA) rather than testing whether factor structures previously determined for the MPNS hold in our sample (36).

We first assessed item distributions for all MPNS-W items to identify items with minimal variation and check for missingness using Stata v16.1 (StataCorp LP, College Station, TX, USA), (Supplementary Table S2). Data were assumed missing at random and pairwise deletion was used for EFA, confirmatory factor analysis (CFA), and measurement invariance models. We created two random-split half samples from our analytic sample. We performed the EFA on the first random-split half sample (N_1_ = 447) and the CFA on the second random-split half sample (N_2_ = 445).

We estimated polychoric correlations to assess relationships between MPNS-W items (37). We then performed the EFA with the weighted least squares (WLSMV) estimator, which is appropriate for items with categorical responses (38), and used oblique rotation (Geomin) as we assumed that factors would be correlated (36). Factors with Eigen values greater than one were examined (Kaiser-Guttman Rule) alongside scree plots, model fit statistics, modification indices, and factor loadings to inform selection of the best fitting model. We only applied model respecifications that were conceptually sound and could be justified using theory and existing evidence.

Items were considered for omission if they had either factor loadings <0.30 or if they cross-loaded significantly on several factors (36). Items were omitted one at a time (item with the lowest factor loading first) and models were iteratively rerun until the model included no items with low factor loadings or cross-loadings. We report factor loadings and model fit statistics (described below) for the identified solution.

#### 2.4.2 Confirmatory factor analysis

To test the factor structure identified in the EFA, we performed a CFA (with the WLSMV estimator) on the second random-split half sample (N_2_ = 445). Root mean square error of approximation (RMSEA) was used as a measure of absolute model fit and the comparative fit index (CFI), Tucker–Lewis index (TLI), and standardized root mean square residual (SRMR) as measures of relative model fit. For RMSEA, values <0.08 indicate adequate fit and values <0.05 indicate good fit (39). For CFI and TLI, values >0.90 indicate adequate fit while values >0.95 indicate good fit and for SRMR, values <0.08 indicate good model fit (40–42). While measures of *χ*^2^ can be sensitive to sample size (40), we include them for completeness. According to the Kline method, a good absolute model fit is indicated by a non-significant *χ*^2^ or a ratio of *χ*^2^ to degrees of freedom (df) that is less than 3:1 (34).

#### 2.4.3 Measurement invariance

When a measure has measurement invariance, it means that the construct it is measuring has the same meaning across the tested conditions (e.g., time of measurement, population, etc.) (43). We test for measurement invariance across the two study settings (Kenya and Nepal). We first assessed single-group solutions by applying the factor solution from the final CFA model to conduct separate CFAs for Kenya (*N* = 565) and Nepal (*N* = 327). We tested for measurement invariance between countries by adding equivalence constraints in a stepwise fashion starting with testing for equal form (configural invariance) to determine whether factor structures were equivalent between countries (44). We added constraints to test for equivalence of factor loadings (metric invariance) and subsequently further constrained item thresholds to be equivalent between countries in order to test for equal indicator intercepts (scalar invariance) (44). Chi-squared difference testing and established thresholds for change in model fit indices (*Δ*CFI ≤.01, *Δ*RMSEA ≤.015, *Δ*SRMR ≤0.03) were used to compare the metric model to the configural model and the scalar model to the metric model (44, 45). The EFA, CFA, and tests of measurement invariance were conducted using Mplus version 8.6 (Muthén & Muthén, Los Angeles, CA, USA).

#### 2.4.4 Internal consistency

Using the factor structure from the final CFA, we calculated scale and sub-scale scores as means of the relevant items such that scores range from zero to three. We then assessed the internal consistency of each of the scale and sub-scale scores for the total sample and the country sub-samples. Internal consistency refers to the degree to which responses are aligned across the items of the scale or sub-scale; if responses are poorly aligned or highly inconsistent, then items may be better assessed individually rather than as part of a scale to measure an overarching construct (34). We estimated Cronbach's alphas and McDonald's omega for each scale and sub-scale to assess internal consistency. The threshold of 0.70 was used to indicate acceptable reliability (23, 46).

#### 2.4.5 Associations with well-being & construct validity

We first assessed distributions of each well-being measure of interest in our total analytic sample and by country. This included wealth (highest wealth quintile vs. any other wealth quintile), psychological well-being (WHO-5 score ≥13 vs. WHO-5 score <13), menstruation-related self-efficacy (“completely confident” vs. “not at all confident”, “slightly confident”, or “very confident”), and work absenteeism (did not miss work vs. missed partial or full day of work). For each binary well-being measure, frequencies were calculated across each category of MPNS-W score (always, more than half the time, less than half the time, and never).

Construct validity refers to the degree to which a tool or score used to measure a latent concept is effective in measuring what it intends to measure (34). These well-being measures were used as hypothesized correlates to confirm MPNS-W scale construct validity. Using Stata v16.1 (StataCorp LP, College Station, TX, USA), we performed binary logistic regressions to test construct validity through hypothesized relationships of scale and sub-scale scores with wealth, psychological well-being, menstruation-related self-efficacy, and work absenteeism. Odds ratios for the latter three outcomes were adjusted for standardized wealth score.

## 3 Results

### 3.1 Participant characteristics

The 892 women who make up the analytic sample ranged in age from 18 to 53 with a mean of about 29 (SD = 6.0) in Kenya and 31 (SD = 7.6) in Nepal (see Table 1). While the plurality of women in Kenya (45%) and the majority of women in Nepal (52%) were married, approximately 40% in both countries were single/never married. The majority of women had received above a secondary education in both Kenya (71%) and Nepal (60%). Women had a variety of job types, the most common of which across both countries were professional/office work (22%), retail (16%), food/lodging (11%), teaching/education (10%), and healthcare (9%). Selling goods at market or other informal settings was much more common in Kenya (14%) than in Nepal (3%). The vast majority of women (92%) were employed throughout the year. In Kenya, women were not screened in 556 of the households approached because there was not a woman at home (218 households) or the woman who was home refused to participate (338 households); an additional 108 women were deemed ineligible during screening (see Participants section above). In Nepal, women were not screened in 266 of the households approached because they were not home (50) or refused to participate (216) and 39 were deemed ineligible during screening. These women were not included in the analytic sample.

### 3.2 Menstruation practices

Most women used disposable sanitary pads (91%) and most often changed menstrual materials in a facility/toilet at their workplace (83%) and disposed of materials inside a toilet stall in the workplace (51%). Using public/shared toilets outside the workplace for changing was also common in Kenya (17%) as was disposing of materials in a rubbish bin outside the toilet stall at the workplace in Nepal (18%).

### 3.3 Dimensionality

**Figure 1:**
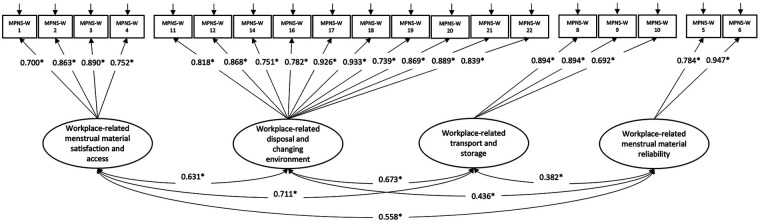
MPNS-W confirmatory factor analysis model (N_2_ = 445) with standardized factor loadings and factor covariances. *p < 0.05; This figure uses standard symbols for the representation of structural equation models (see Kline (34)): ovals represent latent variables (factors), double-headed arrows represent relationship between factors, rectangles represent observed variables (MPNS-W items), single-headed arrows between ovals and rectangles represent relationships between factors and items, single-headed arrows above rectangles represent error terms for each item.

The initial EFA yielded a four-factor solution with acceptable model fit [RMSEA (90% CI) = 0.076 (0.068, 0.084); CFI = 0.971; TLI = 0.952; SRMR = 0.046]. The four factors represented workplace-related menstrual material satisfaction and access (Factor 1), workplace-related disposal and changing environment (Factor 2), workplace-related transport and storage (Factor 3), and workplace-related menstrual material reliability (Factor 4) (see Table 2). However, item 7 (worry about getting more menstrual materials) loaded weakly (0.305) onto Factor 1. This item was thematically similar to item 4 (ability to get more menstrual materials), which had a strong loading on the same factor. Therefore, we excluded item 7. The weak factor loading for item 7 may be related to its negative orientation especially given that all other items on Factor 1 are positively-oriented (47, 48). We reran the EFA without item 7, which yielded the same four-factor solution (sans item 7) and had slightly better model fit [RMSEA (90% CI) = 0.075 (0.066, 0.083); CFI = 0.975; TLI = 0.957; SRMR = 0.044]. CFA confirmed that the four-factor solution fit the data well [RMSEA (90% CI) = 0.077, (0.070, 0.084); CFI = 0.963; TLI = 0.956; SRMR = 0.068]. All items had strong (≥0.7), significant factor loadings. Additionally, each of the four factors was significantly correlated with every other factor, as hypothesized, with factor correlations ranging from 0.4 to 0.7 (see Figure 1).

**Table 2:** MPNS-W standardized factor loadings for random split-half sample EFA (N_1_ = 447) and CFA (N_2_ = 445) models.

Factors and Associated Items		Initial EFA (N_1_ = 447)	Final EFA (N_1_ = 447)	CFA (N_2_ = 445)
Factor 1: Workplace-related menstrual material satisfaction and access				
MPNS-W 1	Were the materials you used to absorb or catch menstrual blood comfortable?	0.659	0.622	0.700
MPNS-W 2	Did you have enough of your menstrual materials to change them as often as you wanted to?	0.581	0.592	0.863
MPNS-W 3	Were you satisfied with your menstrual materials?	0.777	0.815	0.890
MPNS-W 4	Could you get more of your menstrual materials when you needed to (for example, if you needed to purchase materials, retrieve materials from home, or ask someone for materials)?	0.607	0.592	0.752
MPNS-W 7	Were you worried about how you would get more of your menstrual material if you ran out?	0.305	-	-
Factor 2: Workplace-related disposal and changing environment				
MPNS-W 11	Were you able to wash your hands when you wanted to?	0.689	0.472	0.818
MPNS-W 12	Were you able to dispose of your used menstrual materials when you wanted to?	0.725	0.509	0.868
MPNS-W 14	Were you worried about where to dispose of your used menstrual materials?	0.737	0.568	0.751
MPNS-W 16	Were you able to change your menstrual materials when you wanted to?	0.663	0.476	0.782
MPNS-W 17	Were you satisfied with the place you used to change your menstrual materials?	0.990	0.765	0.926
MPNS-W 18	Did you have a clean place to change your menstrual materials?	0.923	0.719	0.933
MPNS-W 19	Were you worried that you would not be able to change your menstrual materials when you needed to?	0.802	0.594	0.739
MPNS-W 20	Were you worried that someone would see you while you were changing your menstrual materials?	0.873	0.789	0.869
MPNS-W 21	Were you worried that someone would harm you while you were changing your menstrual materials?	0.975	0.904	0.889
MPNS-W 22	Were you worried that something else would harm you while you were changing your menstrual materials (e.g., animals, insects, unsafe structure)?	1.004	0.915	0.839
Factor 3: Workplace-related transport and storage				
MPNS-W 8	Did you feel comfortable carrying spare menstrual materials with you to work?	0.903	0.943	0.894
MPNS-W 9	Did you feel comfortable carrying spare menstrual materials to the place where you changed them?	0.698	0.826	0.894
MPNSW-10	Did you have a place to store extra menstrual materials?	0.512	0.529	0.692
Factor 4: Workplace-related menstrual material reliability				
MPNS-W 5	Were you worried that your menstrual materials would allow blood to pass through to your outer garments?	0.804	0.726	0.784
MPNS-W 6	Were you worried that your menstrual materials would move from place while you were wearing them?	0.790	0.810	0.947

Prompt before items: Please think about the last menstrual period you had while working at your main job outside the home.

Geomin rotated loadings for EFA models. All factor loadings are significant at *p* < 0.05.

Initial EFA: Chi-square = 412.770; df = 116; RMSEA (90% CI) = 0.076 (0.068, 0.084); CFI = 0.971; TLI = 0.952; SRMR = 0.046.

Final EFA: Chi-square = 353.816; df = 101; RMSEA (90% CI) = 0.075 (0.066, 0.083); CFI = 0.975; TLI = 0.957; SRMR = 0.044.

CFA: Chi-square = 529.709; df = 146; RMSEA (90% CI) = 0.077 (0.070, 0.084); CFI = 0.963; TLI = 0.956; SRMR = 0.068.

We then conducted separate CFAs (i.e., single-group solutions) for Kenya (*N* = 565) and Nepal (*N* = 327) using the final factor solution (see Table 3). These revealed good relative model fit in Kenya (CFI = 0.944; TLI = 0.935; SRMR = 0.074) and Nepal (CFI = 0.984; TLI = 0.981; SRMR = 0.069); the RMSEA (absolute model fit) in Kenya was slightly above the acceptable threshold [RMSEA (90% CI) = 0.093, (0.087, 0.099)] while in Nepal it was well within the acceptable threshold, but non-significant [RMSEA (90% CI) = 0.051, (0.042, 0.061)]. However, further tests of measurement invariance demonstrate that factor structures, factor loadings, and indicator intercepts were equivalent between countries (metric vs. configural: *Δ*CFI = 0.006, *Δ*RMSEA = 0.008, *Δ*SRMR = 0.003; scalar vs. metric: *Δ*CFI = 0.007, *Δ*RMSEA = 0.002, *Δ*SRMR = 0.002).

**Table 3:** MPNS-W tests of measurement invariance by country.

Model	X^2^	df	X^2^ diff	*Δ*df	RMSEA (90% CI)	CFI	TLI	SRMR	*Δ*RMSEA	*Δ*CFI	*Δ*TLI	*Δ*SRMR
Single-group solutions												
Kenya (*n* = 565)	858.056*	146	–	–	0.093* (0.087, 0.099)	0.944	0.935	0.074	–	–	–	–
Nepal (*n* = 327)	272.005*	146	–	–	0.051 (0.042, 0.061)	0.984	0.981	0.069	–	–	–	–
Measurement invariance (*n* = 892)												
Equal form (Configural)	1,047.389*	292	–	–	0.076* (0.071, 0.081)	0.964	0.958	0.072	–	–	–	–
Equal factor loadings (Metric)	940.398*	307	32.930*	15	0.068* (0.063, 0.073)	0.970	0.966	0.075	0.008	0.006	0.008	0.003
Equal indicator intercepts (Scalar)	1,137.648*	360	309.754*	53	0.070* (0.065, 0.074)	0.963	0.965	0.077	0.002	0.007	0.001	0.002

Model fit thresholds: X^2^ *p* > 0.05, X^2^ diff *p* > 0.05, RMSEA <0.08, CFI >0.95, TLI >0.95, SRMR <0.08, *Δ*CFI ≤.01, *Δ*RMSEA ≤.015, *Δ*SRMR ≤0.03.

* *p* < 0.05.

### 3.4 Scores & internal consistency

Mean MPNS-W scores were high overall (2.6 out of a possible score of 3) and across the sub-scales, ranging from 2.2 to 2.6 (see Table 4). This was consistent with the observed responses to individual scale items, for example 71% of women were “always” satisfied with their menstrual materials; however, this still leaves nearly 30% of women who did not always have this need met (see Supplementary Table S2 for all individual item response frequencies). To confirm that these high scores were representative of the context, we examined women's access to menstrual facilities and materials in each country. These contextual measures confirm the patterns observed in the MPNS-W scores whereby both countries have higher than expected access to facilities and materials and—according to most measures we assessed—a slightly higher proportion of women in Nepal than women in Kenya have access. For example, 81% of women in Kenya and 83% of women in Nepal reported that their workplace had designated sanitation facilities for workers to use; similarly, 96% of women in Kenya and 98% of women in Nepal reported that they were able to obtain the quantity of menstrual materials they desired during their last menstrual period (Supplementary Table S3).

**Table 4:** MPNS-W categorical scale score and well-being measures, by country.

MPNS-W categorical scale score	Total n, (%)	Highest wealth quintile n, (%)	Psychological well-being WHO-5 ≥ 13/25 n, (%)	Did not miss work because of last menstrual period n, (%)	Completely confident managing menstruation at work n, (%)
Overall (*n* = 892)	–	174, (19.7%)	609, (68.7%)	769 (86.3%)	167, (18.8%)
Always *(scale score = 3)*	137, (15.4%)	27, (20.3%)	115, (83.9%)	128, (93.4%)	46, (33.6%)
More than half the time *(scale score <3 & ≥2)*	667, (74.9%)	141, (21.2%)	454, (68.4%)	582, (87.3%)	118, (17.7%)
Less than half the time *(scale score <2 & ≥1)*	76, (8.5%)	6, (7.9%)	37, (49.3%)	51, (68.0%)	3, (4.0%)
Never *(scale score <1)*	10, (1.1%)	0, (0%)	3, (30.0%)	6, (60.0%)	0, (0%)
Kenya (*n* = 565)	–	111, (19.7%)	330, (58.9%)	473, (84.0%)	42, (7.5%)
Always *(scale score = 3)*	75, (13.3%)	19, (25.7%)	58, (77.3%)	67, (89.3%)	12, (16.0%)
More than half the time *(scale score <3 & ≥2)*	420, (74.5%)	86, (20.5%)	242, (58.0%)	360, (85.7%)	30, (7.1%)
Less than half the time *(scale score <2 & ≥1)*	59, (10.5%)	6, (10.2%)	27, (46.6%)	40, (69.0%)	0, (0%)
Never *(scale score <1)*	10, (1.8%)	0, (0%)	3, (30.0%)	6, (60.0%)	0, (0%)
Nepal (*n* = 327)	–	63, (19.6%)	279, (85.6%)	294, (90.2%)	125, (38.6%)
Always *(scale score = 3)*	62, (19.0%)	8, (13.6%)	57, (91.9%)	61, (98.4%)	34, (54.8%)
More than half the time *(scale score <3 & ≥2)*	247, (75.8%)	55, (22.4%)	212, (85.8%)	222, (89.9%)	88, (35.9%)
Less than half the time *(scale score <2 & ≥1)*	17, (5.2%)	0, (0%)	10, (58.8%)	11, (64.7%)	3, (17.7%)
Never *(scale score <1)*	0, (0%)	–	–	–	–

Scale score missing 2 (1 in Kenya and 1 in Nepal); Wealth quintile missing 1 in Kenya, 4 in Nepal; WHO-5 score missing 4 in Kenya; Confidence in managing menstruation (completely confident vs. very confident, slightly confident, or not at all confident) missing 2 in Nepal; Missed work because of last menstrual period missing 1 in Kenya.

Women in Nepal had statistically significantly higher scale and sub-scale scores for *workplace-related menstrual material satisfaction and access* as well as for *workplace-related disposal and changing environment*, indicating that women in Nepal had more positive menstrual experiences in the workplace than women in Kenya, driven especially by these two dimensions. However, women in Nepal had statistically significantly lower *workplace-related menstrual material reliability* scores than women in Kenya. In both countries, *workplace-related menstrual material reliability* was the sub-scale with the lowest mean indicating that it was the area in which women had the greatest unmet need. Overall, the scale and sub-scale scores achieved acceptable reliability according to McDonald's omega. In Nepal, the *workplace-related transport and storage* sub-scale had poor reliability (0.52) according to Cronbach's alpha and borderline acceptable reliability according to McDonald's omega (0.67), which may be related to the small number of items (3) in this factor (31). The sub-scales should not be used in isolation.

### 3.5 Categorical MPNS-W scores and well-being measures

In both countries, a higher proportion of women “achieved” each well-being measure (e.g., were “completely confident”) with each MPNS-W response category from *never* having needs met to *always* having needs met; the only exception was wealth (see Table 4). Among women whose needs were always met (i.e., whose mean MPNS-W scores were 3), 77% in Kenya and 92% in Nepal had “positive” psychological well-being. The proportion of women who did not miss work because of their last menstrual period was also higher in Nepal at 90% compared to 84% in Kenya; among women whose needs were always met, 89% in Kenya and 98% in Nepal did not miss work compared to 60% of women whose needs were never met in Kenya and 65% of women whose needs were met less than half the time in Nepal. Only 8% of women in Kenya reported being completely confident managing menstruation at work compared to 39% in Nepal. Among women whose needs were always met, 16% in Kenya and 55% in Nepal felt completely confident; none of the women in Kenya whose needs were never met or met less than half the time felt completely confident managing menstruation while 18% of women in Nepal whose needs were met less than half the time felt completely confident.

### 3.6 Construct validity

We tested construct validity by assessing bivariate relationships between scale and sub-scale scores and hypothesized correlates, adjusted for standardized wealth score (Table 5). The unadjusted association between MPNS-W scale score and wealth revealed that for every one-point increase in MPNS-W scale score (0–3), the odds of being in the highest wealth quintile [OR = 1.55, 95% CI = (1.03, 2.34)] were two times greater. The adjusted associations between MPNS-W scale score and the remaining three well-being outcomes showed that for every one point increase in MPNS-W scale score, the odds of having positive well-being [OR = 2.46, 95% CI = (1.79, 3.39)] were two times greater, the odds of not missing work because of the last menstrual period [OR = 2.93, 95% CI = (2.01, 4.27)] were three times greater, and the odds of being completely confident managing menstruation at work [OR = 8.00, 95% CI = (4.10, 15.61)] were eight times greater. The relationship between MPNS-W score and wealth appeared to be driven by the *workplace-related disposal and changing environment* sub-scale scores. In both countries, higher MPNS-W score as well as higher sub-scale scores for *workplace-related menstrual material satisfaction and access* and *workplace-related disposal and changing environment* were significantly associated with increased odds of having positive psychological well-being. Higher MPNS-W scores were significantly associated with increased odds of women attending work during their last menstrual period; this was true across all scale and sub-scale scores in both countries with the exception of *workplace-related menstrual material satisfaction and access* in Kenya only. In both countries, MPNS-W scores and sub-scale scores for *workplace-related menstrual material satisfaction and access* and *workplace-related menstrual material reliability* were significantly associated with increased odds that women felt “completely confident” managing menstruation in the workplace, as were sub-scale scores for *workplace-related disposal and changing environment* in Kenya only.

**Table 5:** MPNS-W scale and sub-scale score internal consistency and construct validity, overall and by country.

MPNS-W scale or sub-scale	Mean	SD	*α*	*Ω*	Highest wealth quintile		Psychological well-being WHO-5 ≥ 13/25		Did not miss work because of last menstrual period		Completely confident managing menstruation at work	
					OR (95% CI)	*p*-value	OR (95% CI)	*p*-value	OR (95% CI)	*p*-value	OR (95% CI)	*p*-value
Overall (*n* = 892)												
Scale score	2.58	0.45	0.90	0.91	1.55 (1.03, 2.34)	0.037	2.46 (1.79, 3.39)	<0.001	2.93 (2.01, 4.27)	<0.001	8.00 (4.10, 15.61)	<0.001
Factor 1: Workplace-related menstrual material satisfaction and access	2.63	0.49	0.81	0.82	0.94 (0.68, 1.32)	0.735	1.95 (1.46, 2.59)	<0.001	1.52 (1.06, 2.18)	0.022	4.26 (2.51, 7.23)	<0.001
Factor 2: Workplace-related disposal and changing environment	2.62	0.54	0.90	0.91	2.06 (1.38, 3.09)	<0.001	2.34 (1.79, 3.06)	<0.001	2.20 (1.62, 2.98)	<0.001	7.88 (3.93, 15.80)	<0.001
Factor 3: Workplace-related transport and storage	2.63	0.62	0.68	0.73	1.01 (0.77, 1.32)	0.969	1.28 (1.02, 1.60)	0.033	1.83 (1.40, 2.40)	<0.001	1.07 (0.80, 1.43)	0.645
Factor 4: Workplace-related menstrual material reliability	2.15	0.82	0.76	–	0.94 (0.77, 1.14)	0.522	1.02 (0.85, 1.21)	0.844	1.70 (1.38, 2.11)	<0.001	1.89 (1.46, 2.45)	<0.001
Kenya (*n* = 565)												
Scale score	2.51*	0.49	0.91	0.91	1.44 (0.91, 2.28)	0.123	1.78 (1.25, 2.54)	0.001	2.36 (1.55, 3.59)	<0.001	11.18 (3.11, 40.12)	<0.001
Factor 1: Workplace-related menstrual material satisfaction and access	2.59*	0.53	0.84	0.85	0.87 (0.59, 1.27)	0.461	1.52 (1.10, 2.10)	0.011	1.08 (0.71, 1.64)	0.730	3.10 (1.28, 7.52)	0.012
Factor 2: Workplace-related disposal and changing environment	2.50*	0.60	0.90	0.90	1.87 (1.21, 2.88)	0.005	1.73 (1.29, 2.32)	<0.001	1.90 (1.35, 2.67)	<0.001	9.69 (2.69, 34.98)	0.001
Factor 3: Workplace-related transport and storage	2.63	0.62	0.79	0.80	0.86 (0.63, 1.18)	0.351	1.21 (0.93, 1.59)	0.159	1.88 (1.37, 2.58)	<0.001	1.56 (0.81, 3.02)	0.186
Factor 4: Workplace-related menstrual material reliability	2.19*	0.80	0.67	–	1.04 (0.80, 1.36)	0.760	0.99 (0.80, 1.22)	0.913	1.88 (1.45, 2.44)	<0.001	3.17, (1.68, 5.97)	<0.001
Nepal (*n* = 327)												
Scale score	2.69*	0.33	0.86	0.88	2.35 (0.87, 6.36)	0.092	3.65 (1.64, 8.13)	0.001	6.07 (2.33, 15.81)	<0.001	4.39 (1.81, 10.62)	0.001
Factor 1: Workplace-related menstrual material satisfaction and access	2.72*	0.40	0.74	0.75	1.26 (0.61, 2.60)	0.528	3.31 (1.67, 6.57)	0.001	4.26 (1.97, 9.21)	<0.001	4.95 (2.35, 10.42)	<0.001
Factor 2: Workplace-related disposal and changing environment	2.83*	0.34	0.88	0.90	10.99 (1.9, 63.5)	0.007	2.45 (1.16, 5.16)	0.019	3.72 (1.54, 8.99)	0.003	1.99 (0.88, 4.47)	0.096
Factor 3: Workplace-related transport and storage	2.62	0.60	0.52	0.67	1.41 (0.85, 2.35)	0.183	1.74 (1.10, 2.75)	0.018	1.79 (1.07, 2.99)	0.027	0.97 (0.67, 1.42)	0.893
Factor 4: Workplace-related menstrual material reliability	2.08*	0.86	0.89	–	0.80 (0.59, 1.09)	0.162	1.38 (0.97, 1.96)	0.073	1.52 (1.02, 2.25)	0.037	2.24 (1.61, 3.10)	<0.001

Logistic regressions on psychological well-being, did not miss work, and completely confident are each adjusted for standardized wealth score; *Significant difference between countries at *p* < 0.05; *Ω* requires at least three items to estimate; Scale score missing 2 (1 in Kenya and 1 in Nepal), Factor 2 missing 1 in Nepal, Factor 4 missing 1 in Kenya for women who had missing values for more than one item in a sub-scale; Wealth quintile/standardized wealth score missing 1 in Kenya, 4 in Nepal; WHO-5 score missing 4 in Kenya; Confidence in managing menstruation (completely confident vs. very confident, slightly confident, or not at all confident) missing 2 in Nepal; Missed work because of last menstrual period missing 1 in Kenya.

## 4 Discussion

We validated the MPNS-W and assessed relationships between MPNS-W scores and well-being outcomes among adult women working outside the home in urban areas of Nairobi, Kenya and Kathmandu, Nepal using cross-sectional survey data from the Advancement of Metrics for Menstrual Health and Hygiene in the Workplace activity under the WASHPaLS project. Rigorous measurement validation analyses confirmed the MPNS-W's factor structure, internal consistency, and construct validity. Binary logistic regression demonstrated that women with higher MPNS-W scores had significantly higher odds of reporting positive psychological well-being, not missing work because of the last menstrual period, and being completely confident managing menstruation at work, even after controlling for wealth.

### 4.1 MPNS-W factor structure & validation

The final, validated tool, or the MPNS-W, is a 19-item, multidimensional scale with the following four factors: *workplace-related menstrual material satisfaction and access, disposal and changing environment, transport and storage*, and *menstrual material reliability*. The EFA and CFA using data from both countries yielded models with good model fit according to relative model fit statistics (CFI, TLI, and SRMR) and absolute model fit statistics (RMSEA). In the final CFA, all items had strong and significant factor loadings and each of the four factors was significantly correlated with every other factor, further validating the final factor structure. Tests of measurement invariance confirmed that the factor structure was equivalent across the two country settings. Evidence for construct validity included significant bivariate relationships between MPNS-W score and the hypothesized correlates determined *a priori* (WHO-5 score, work absenteeism, and confidence managing menstruation at work)*,* even after controlling for wealth. The findings for construct validity were largely consistent with Hennegan et al. (11) who found that the workplace needs factor of the MPNS adapted for adult working women in Uganda was significantly associated with WHO-5 score and confidence managing menstruation at work but was not significantly associated with work absenteeism.

Internal consistency statistics, which assess the degree to which respondents' answers agree across the tool or across items within the same factor, were within an acceptable range with the exception of the internal consistency coefficients for *workplace-related transport and storage.* This sub-scale had poor reliability according to Cronbach's alpha and borderline acceptable reliability according to McDonald's omega. Cronbach's alpha has been shown to consistently underestimate reliability, particularly for scales with ordinal items (49–51). Low internal consistency is also common when there are few items representing a factor (31, 36); *workplace-related transport and storage* has the smallest number of items of any of the three factors for which a McDonald's omega is available. More work is necessary to expand this sub-scale before it could be used in isolation. These findings demonstrate that the final 19-item, four-factor scale is both valid and reliable among our study populations in urban Kenya and Nepal, and that scale scores can be validly compared across the two country settings.

The workplace-specific MPNS-W or the multi-location MPNS may be more or less appropriate depending on study aims and context. The MPNS-W has very practical and distinct sub-scales that are less complicated by relationships with the home environment and allow for easier identification and prioritization of the areas of greatest need. Imposing a location-specific focus to the tool (rather than asking about both the work and home environment in different items on the same tool) yielded factors and sub-scale scores that reflect unmet needs in a specific category (e.g., menstrual materials, changing/disposing location, storage and transport). The MPNS captures menstrual experiences across locations and has factors/scores that lump together various categories of needs but provides a more summative picture of the degree of unmet need/insecurity by location. This may be useful for researchers or practitioners interested in the impact of menstrual experiences on more encompassing outcomes such as general health or psychological well-being. However, we were able to observe a significant association even between the location-specific MPNS-W and psychological well-being in our study sample.

### 4.2 MPNS-W scores & relationships with well-being

The MPNS-W scores were high in both countries (2.51 in Kenya and 2.69 in Nepal out of a total possible score of 3) yet similar to what was found using the MPNS among adult working women in Uganda (23). Scale scores and all but one sub-scale score (*workplace-related transport and storage*) were higher in Nepal than in Kenya. This was representative of the context and consistent with women's access to menstrual facilities and materials in each country (Supplementary Table S3). Hennegan et al. found higher MPNS scores (average of 2.47) among adult working women than among adolescent girls (1.82) in Uganda, which they hypothesized was reflective of urban working women having “greater access to resources and more experience managing menstruation” (21, 23).

MPNS-W scores were significantly associated with each of the tested well-being measures that were hypothesized *a priori* to be correlates of menstrual needs and experience based on existing evidence (1, 10, 12, 23, 25, 52–56). We hypothesized that women with more wealth—who have greater access to resources—would have more of their menstrual needs met (1, 23, 52, 54). Our finding that higher wealth quintile was associated with having more menstrual needs met aligns with findings from a systematic review and meta-analysis in Nepal, which found that economic status was a key factor impacting menstrual health (55) and findings from a cohort of adolescent girls in Nairobi County who reported that the most common barrier to accessing menstrual materials was financial (56). Having more menstrual needs met was also strongly associated with better psychological well-being, even after controlling for wealth, which is consistent with findings from other studies that have found that unmet menstrual needs can increase stress, anxiety, depression (1, 10, 23) and social isolation (12). As with findings from adult working women in Uganda (23), having more menstrual needs met was associated with feeling completely confident managing menstruation at work.

Work absenteeism, of all well-being measures investigated, was most consistently associated with sub-scale scores across all four MPNS-W factors. Regardless of the type or category of menstrual need, having needs met was significantly associated with not having missed work during the woman's last menstrual period, even after adjusting for wealth. Other studies similarly found that unmet menstrual needs can limit women's participation in employment and ability to perform well in the workplace (1, 25). However, the workplace needs factor of the MPNS adapted for adult working women in Uganda was not significantly associated with work absenteeism (23). Hennegan et al. (11) explain that this may have been due to missing cases “where those absent from the workplace were unable to report on their experiences at work”; our sampling strategy of recruiting working women at their homes as opposed to at their workplaces overcomes this limitation and allowed us to identify an important and consistent relationship between unmet menstruation-related needs across the MPNS-W factors and work absenteeism.

### 4.3 Strengths & limitations

This study makes an important contribution to advancing methodologies for measuring women's menstrual needs and experiences. We conducted multiple tests of validity (EFA, CFA, tests of measurement invariance, and associations with *a priori* hypothesized correlates) and reliability (estimates for Cronbach's alpha and McDonald's omega) using best practices in scale development and validation. The MPNS-W also underwent a rigorous review and refinement process (including expert review, cognitive interviewing, and piloting) prior to data collection to ensure content and face validity of each item. By targeting women respondents at their homes instead of their workplaces, we were able to collect data from a sample of women whose menstrual experiences cover a broad variety of workplace environments. However, this also meant that we were unable to make observations of workplace facilities, which would have otherwise helped triangulate study findings, particularly around higher than expected MPNS-W scores. Another important strength of the study is the random selection of participants within the urban study areas, which increases representativeness and external validity of our results. Further research may be required to adapt the MPNS-W to rural settings where job types, socioeconomic status, or educational attainment may differ. We were unable to validate the sub-scales and items related to reuse, washing, and drying of menstrual materials because reusable menstrual materials were used by so few women in our sample.

### 4.4 Implications & recommendations

Menstrual experiences are multidimensional, necessitating programs that address and tools that measure the breadth and depth of menstrual needs and practices. Even after modifications to the MPNS to focus exclusively on the workplace environment, the final validated scale consisted of four factors covering a variety of topics/experiences from menstrual material satisfaction, access, and reliability to transport and storage of menstrual materials to features of and experiences with facilities for changing and disposal. The fact that the workplace-specific MPNS-W retained a multidimensional factor structure speaks to the complexity of menstrual experiences. The MPNS-W is an important advancement in measurement in the sector that moves us beyond singular indicators or facilities observation checklists toward a tool that reflects the larger constellation of women's menstrual experiences. While conventional wisdom may suggest that researchers or program evaluators should evaluate the outcomes that are most closely linked to the exposure of interest or that are directly targeted by the intervention, this ignores how the exposure or intervention activities may interact with other parts of the menstrual experience. For example, provision of disposable sanitary pads may directly improve access to or satisfaction with menstrual materials, but tools or indicators that measure only these aspects of the menstrual experience ignore the fact that provision of disposable sanitary pads could also introduce new disposal challenges and impact changing experiences.

Furthermore, it is clear from our investigation of the associations between MPNS-W scores and well-being measures that scale scores are the most powerful in predicting outcomes of interest—further evidence that data for all of the MPNS-W sub-scales should be collected and evaluated together to capture multidimensional menstrual experiences. While sub-scales should not be used in isolation (especially the *workplace-related transport and storage* sub-scale, which had a suboptimal internal consistency score), they do provide useful information when examined relative to one another. Sub-scale scores could be used to find the area(s) of greatest need or to prioritize intervention components while scale scores are likely best for assessing relationships with antecedents or outcomes of interest. Future research should test the use of the MPNS-W scores in measuring the impact of an intervention (i.e., change in MPNS-W scores pre/post intervention or difference in MPNS-W scores between treatment and control groups); while this has not yet been done with either the MPNS-W nor with the earlier iterations of the MPNS, this is an important potential use for the tool that should be explored by researchers and/or program evaluators.

## Data Availability

Publicly available datasets were analyzed in this study. This data can be found here: The datasets analyzed during the current study are publicly available at https://doi.org/10.6084/m9.figshare.31424900.
