# Supplementary material for: Menstrual practice needs scale for the workplace: validation and associations with well-being among adult women in Kathmandu, Nepal and Nairobi, Kenya
Source: Front Glob Womens Health. 2026 Apr 22;7:1693187. doi: 10.3389/fgwh.2026.1693187 (PMC13144123; doi:10.3389/fgwh.2026.1693187)
Supplement: Supplementary file 1 [file Table1.docx]

| **Supplementary Table A.** Menstrual Practice Needs Scale with Revisions for the Workplace | | | |
| --- | --- | --- | --- |
| **Item** | **MPNS Item (Hennegan et al., 2022)** | **MPNS-W Item** | **Reason for Modification** |
| MPNS-W 1 | Were your menstrual materials comfortable? | Were the materials you used to absorb or catch menstrual blood comfortable? | Slight wording changes to improve clarity for participant |
| MPNS-W 2 | Did you have enough of your menstrual materials to change them as often as you wanted to? | No change | N/A |
| MPNS-W 3 | Were you satisfied with the cleanliness of your menstrual materials? | Were you satisfied with your menstrual materials? | Removed reference to “cleanliness” based on cognitive interviews; most women did not use reusable menstrual materials |
| MPNS-W 4 | Could you get more of your menstrual materials when you needed to? | Could you get more of your menstrual materials when you needed to (for example, if you needed to purchase materials, retrieve materials from home, or ask someone for materials)? | During cognitive interviews, participants were unsure if the question referred to purchasing or retrieving materials; modification clarifies that question refers to both |
| MPNS-W 5 | Were you worried that your menstrual materials would allow blood to pass through to your outer garments? | No change |  |
| MPNS-W 6 | Were you worried that your menstrual materials would move from place while you were wearing them? | No change |  |
| MPNS-W 7 | Were you worried about how you would get more of your menstrual material if you ran out? | No change |  |
| MPNS-W 8 | Did you feel comfortable carrying spare menstrual materials with you outside your home? | Did you feel comfortable carrying spare menstrual materials with you to work? | Specified ‘work’ given workplace focus of the revised scale |
| MPNS-W 9 | Did you feel comfortable carrying menstrual materials to the place where you changed them? | Did you feel comfortable carrying spare menstrual materials to the place where you changed them? | Slight wording changes to improve clarity for participant |
| MPNS-W 10 | Did you feel comfortable storing [keeping] your leftover or cleaned menstrual materials until your next period? | Did you have a place to store extra menstrual materials? | Storing materials at work until the next period was not common practice; women could not assess 'comfort' if they did not have access to a place for storing materials |
| MPNS-W 11 | Were you able to wash your hands when you wanted to? | No change |  |
| MPNS-W 12 | Were you able to immediately dispose of your used menstrual materials? | Were you able to dispose of your used menstrual materials when you wanted to? | Original item had reliability issues in Hennegan et al., 2022 |
| MPNS-W 13 | Were you able to dispose of your used materials in the way that you wanted to? | Excluded from survey | Caused confusion in cognitive interviews |
| MPNS-W 14 | Were you worried about where to dispose of your used menstrual materials? | No change |  |
| MPNS-W 15 | Were you concerned that others would see your used menstrual materials in the place you disposed of them? | Excluded from survey | Not clear/relevant to respondents during cognitive interviews. |
| MPNS-W 16 | When at home, were you able to change your menstrual materials when you wanted to? | Were you able to change your menstrual materials when you wanted to? | Revised version refers to workplace rather than home environment |
| MPNS-W 17 | When at home, were you satisfied with the place you used to change your menstrual materials? | Were you satisfied with the place you used to change your menstrual materials? | Revised version refers to workplace rather than home environment |
| MPNS-W 18 | When at home, did you have a clean place to change your menstrual materials? | Did you have a clean place to change your menstrual materials? | Revised version refers to workplace rather than home environment |
| MPNS-W 19 | When at home, were you worried that you would not be able to change your menstrual materials when you needed to? | Were you worried that you would not be able to change your menstrual materials when you needed to? | Revised version refers to workplace rather than home environment |
| MPNS-W 20 | When at home, were you worried that someone would see you while you were changing your menstrual materials? | Were you worried that someone would see you while you were changing your menstrual materials? | Revised version refers to workplace rather than home environment |
| MPNS-W 21 | When at home, were you worried that someone would harm you while you were changing your menstrual materials? | Were you worried that someone would harm you while you were changing your menstrual materials? | Revised version refers to workplace rather than home environment |
| MPNS-W 22 | When at home, were you worried that something else would harm you while you were changing your menstrual materials (eg, animals, unsafe structure) | Were you worried that something else would harm you while you were changing your menstrual materials (e.g., animals, insects, unsafe structure)? | Revised version refers to workplace rather than home environment |
| MPNS-W 23 | When at work, were you able to change your menstrual materials when you wanted to? | Excluded from survey | Redundancy with MPNS-W 16 in revised version |
| MPNS-W 24 | When at work, were you satisfied with the place you used to change your menstrual materials? | Excluded from survey | Redundancy with MPNS-W 17 in revised version |
| MPNS-W 25 | When at work, did you have a clean place to change your menstrual materials? | Excluded from survey | Redundancy with MPNS-W 18 in revised version |
| MPNS-W 26 | When at work, were you worried that you would not be able to change your menstrual materials when you needed to? | Excluded from survey | Redundancy with MPNS-W 19 in revised version |
| MPNS-W 27 | When at work, sere you worried that someone would see you while you were changing your menstrual materials? | Excluded from survey | Redundancy with MPNS-W 20 in revised version |
| MPNS-W 28 | When at work, were you worried that someone would harm you while you were changing your menstrual materials? | Excluded from survey | Redundancy with MPNS-W 21 in revised version |
| MPN31S-W a | New item | Do you ever use reusable menstrual materials while working? | MPNS-W 29-38 and MPNS-W 44 only asked if yes to MPNS-W 31a |
| MPNS-W 29 | Did you have enough water to soak or wash your menstrual materials? | No change |  |
| MPNS-W 30 | Did you have access to a basin or bucket to soak or wash your menstrual materials whenever you needed it? | No change |  |
| MPNS-W 31 | Were you able to wash your menstrual materials whenever you wanted to? | Were you able to wash your menstrual materials if you needed to? | Experts suggested 'need' more relevant, meaningful than 'want' in workplace context; aligns with Menstrual Health Indicators |
| MPNS-W 32 | Did you have enough soap to wash your menstrual materials? | No change |  |
| MPNS-W 33 | Were you able to dry your materials when you wanted to? | No change |  |
| MPNS-W 34 | Were you worried that someone would see you while you were washing your menstrual materials? | No change |  |
| MPNS-W 35 | Were you worried that your menstrual materials would not be dry when you needed them? | No change |  |
| MPNS-W 36 | Were you worried that others would see your menstrual materials while they were drying? | No change |  |

| **Supplementary Table B.** Distribution and missingness for MPNS-W items | | | | | | |
| --- | --- | --- | --- | --- | --- | --- |
| Item | Never | Less than half the time | More than half the time | Always | NA | Chose not to answer |
| **All women (N=940)** – excluding those who never changed menstrual materials at work | | | | | | |
| MPNS-W 1 | 11, 1.2% | 51, 5.4% | 270, 28.7% | 607, 64.6% | 0 | 1, 0.1% |
| MPNS-W 2 | 6, 0.6% | 43, 4.6% | 230, 24.5% | 661, 70.3% | 0 | 0 |
| MPNS-W 3 | 4, 0.4% | 38, 4.0% | 229, 24.4% | 668, 71.1% | 0 | 1, 0.1% |
| MPNS-W 4 | 14, 1.5% | 56, 6.0% | 210, 22.3% | 660, 70.2% | 0 | 0 |
| MPNS-W 5 | 339, 36.1% | 397, 42.2% | 109, 11.6% | 93, 9.9% | 0 | 2, 0.2% |
| MPNS-W 6 | 448, 47.7% | 318, 33.8% | 105, 11.2% | 68, 7.2% | 0 | 1, 0.1% |
| MPNS-W 7 | 586, 62.3% | 220, 23.4% | 69, 7.3% | 64, 6.8% | 0 | 1, 0.1% |
| MPNS-W 8 | 23, 2.5% | 43, 4.6% | 107, 11.4% | 765, 81.4% | 2, 0.2% | 0 |
| MPNS-W 9 | 39, 4.2% | 78, 8.3% | 147, 15.6% | 674, 71.7% | 2, 0.2% | 0 |
| MPNS-W 10 | 73, 7.8% | 37, 3.9% | 101, 10.7% | 724, 77.0% | 0 | 5, 0.5% |
| MPNS-W 11 | 20, 2.1% | 73, 7.8% | 146, 15.5% | 701, 74.6% | 0 | 0 |
| MPNS-W 12 | 30, 3.2% | 63, 6.7% | 127, 13.5% | 678, 72.1% | 41, 4.4%** | 1, 0.1% |
| MPNS-W 14 | 630, 67.0% | 117, 12.5% | 61, 6.5% | 96, 10.2% | 35, 3.7%** | 1, 0.1% |
| MPNS-W 16 | 22, 2.3% | 84, 8.9% | 203, 21.6% | 631, 67.1% | 0 | 0 |
| MPNS-W 17 | 47, 5.0% | 81, 8.6% | 175, 18.6% | 637, 67.8% | 0 | 0 |
| MPNS-W 18 | 29, 3.1% | 98, 10.4% | 159, 16.9% | 653, 69.5% | 0 | 1, 0.1% |
| MPNS-W 19 | 579, 61.6% | 248, 26.4% | 69, 7.3% | 44, 4.7% | 0 | 0 |
| MPNS-W 20 | 808, 86.0% | 87, 9.3% | 24, 2.6% | 21, 2.2% | 0 | 0 |
| MPNS-W 21 | 852, 90.6% | 56, 6.0% | 19, 2.0% | 12, 1.3% | 0 | 1, 0.1% |
| MPNS-W 22 | 843, 89.7% | 54, 5.7% | 20, 2.1% | 23, 2.5% | 0 | 0 |
| **Sub-sample of only women who reuse materials (N = 75)** | | | | | | |
| MPNS-W 29* | 8, 10.7% | 4, 5.3% | 4, 5.3% | 24, 32.0% | 33, 44.0% | 2, 2.7% |
| MPNS-W 30* | 10, 13.3% | 5, 6.7% | 4, 5.3% | 21, 28.0% | 33, 44.0% | 3, 4.0% |
| MPNS-W 31* | 8, 10.7% | 4, 5.3% | 9, 12.0% | 22, 29.3% | 30, 40.0% | 2, 2.7% |
| MPNS-W 32* | 5, 6.7% | 4, 5.3% | 4, 5.3% | 25, 33.3% | 35, 46.7% | 2, 2.7% |
| MPNS-W 33* | 7, 9.3% | 5, 6.7% | 5, 6.7% | 15, 20.0% | 40, 53.3% | 3, 4.0% |
| MPNS-W 34* | 18, 24.0% | 7, 9.3% | 3. 4.0% | 7, 9.3% | 37, 49.3% | 3, 4.0% |
| MPNS-W 35* | 16, 21.3% | 3, 4.0% | 5, 6.7% | 7, 9.3% | 41, 54.7% | 3, 4.0% |
| MPNS-W 36* | 15, 20.0% | 4, 5.3% | 1, 1.3% | 10, 13.3% | 41, 54.7% | 4, 5.3% |
| *Items excluded before initial EFA. **42 women who replied ‘NA- Did not dispose of any materials at work’ to either MPNS-W 12 or MPNS-W 14 were excluded from analyses before initial EFA in addition to 6 who replied ‘NA – Did not dispose of any materials at work’ to the question “During your last menstrual period, where did you most often dispose of your used menstrual materials while working outside the home?” | | | | | | |

| **Supplementary Table C.** Access to facilities and materials for managing menstruation, by country | | | | | | |
| --- | --- | --- | --- | --- | --- | --- |
|  | **All (N=892)** | | **Kenya (N=565)** | | **Nepal (N=327)** | |
| **Facilities** | | | | | | |
| Workplace has private facilities for women to bathe/wash themselves or wash reusable materials | 428 | 48.2% | 214 | 38.1% | 214 | 65.6% |
| Workplace has designated sanitation facilities for workers to use | 729 | 81.9% | 458 | 81.4% | 271 | 82.9% |
| If yes, type of sanitation facilities available (select all that apply) | n=729 | | n=458 | | n=271 | |
| Flush/pour-flush | 660 | 90.5% | 423 | 92.4% | 237 | 87.5% |
| Dry toilet | 75 | 10.3% | 58 | 12.7% | 17 | 6.3% |
| Other | 18 | 2.5% | 1 | 0.2% | 17 | 6.3% |
| Is the place you most often use to change your menstrual materials while you are working… | | | | | | |
| …separate from men’s facilities?* | 587 | 68.0% | 400 | 73.7% | 187 | 58.4% |
| …structurally private?** | 586 | 96.5% | 268 | 94.7% | 318 | 98.2% |
| …clean? | 805 | 91.6% | 490 | 88.5% | 315 | 96.9% |
| …usually has a wait due to lines or crowding? | 161 | 18.1% | 132 | 23.4% | 29 | 8.9% |
| …lockable from the inside?* | 836 | 96.8% | 519 | 95.2% | 317 | 99.4% |
| …pay per use?* | 106 | 12.2% | 101 | 18.5% | 5 | 1.6% |
| …have a handwashing station?* | 782 | 90.6% | 483 | 88.8% | 299 | 93.7% |
| …with soap and water? (of those with handwashing station) | 679 | 86.3% | 392 | 80.3% | 287 | 96.0% |
| **Materials** | | | | | | |
| Able to obtain desired quantity of menstrual products during last menstrual period | 849 | 96.7% | 538 | 96.1% | 311 | 97.8% |
| Had to choose between paying for menstruation-related needs and other needs of your household during last menstrual period | 180 | 20.3% | 165 | 29.4% | 15 | 4.7% |
| Are menstrual materials available where you work? (select all that apply) | | | | | | |
| Yes, for free | 89 | 10.0% | 37 | 6.6% | 1 | 0.2% |
| Yes, for purchase | 268 | 30.1% | 201 | 35.6% | 52 | 15.9% |
| Yes, from a friend | 33 | 3.7% | 6 | 1.1% | 67 | 20.5% |
| No | 518 | 58.1% | 323 | 57.3% | 196 | 59.6% |
| % is out of those who chose to answer: (1 in Kenya and 1 in Nepal didn’t know and 2 in Kenya chose not to answer ‘private facilities for women to bathe/wash’; 1 in Kenya didn’t know and 1 in Kenya chose not answer ‘designated facilities’; 3 in Kenya chose not to answer ‘separate from men’s facilities’; 1 in Kenya and 3 in Nepal chose not to answer ‘structurally private’; 11 in Kenya and 2 in Nepal chose not to answer ‘clean’; 1 in Kenya chose not to answer ‘wait due to lines or crowding’; 1 in Kenya and 1 in Nepal chose not to answer ‘lockable from the inside’; 2 in Kenya and 1 in Nepal chose not to answer ‘handwashing station’; 3 in Kenya and 8 in Nepal said N/A and 2 in Kenya and 1 in Nepal chose not to answer ‘obtain quantity of menstrual products; 2 in Kenya and 4 in Nepal said N/A and 1 in Kenya and 1 in Nepal chose not to answer ‘choose between paying for menstrual-related needs and other needs’; 1 in Kenya chose not to answer ‘materials available where you work’)  *Excluded women who reported that the place they most often changed menstrual materials while working was at their home, at someone else's home, or outdoors  **Out of 283 in Kenya and 324 in Nepal | | | | | | |
